# Knowledge, attitude, and practices towards schistosomiasis among rural population in Yemen

**DOI:** 10.1186/s13071-015-1050-8

**Published:** 2015-08-25

**Authors:** Hany Sady, Hesham M. Al-Mekhlafi, Wahib M. Atroosh, Ahmed K. Al-Delaimy, Nabil A. Nasr, Salwa Dawaki, Mona A. Al-Areeqi, Init Ithoi, Awatif M. Abdulsalam, Kek Heng Chua, Johari Surin

**Affiliations:** Department of Parasitology, Faculty of Medicine, University of Malaya, 50603 Kuala Lumpur, Malaysia; Department of Medical Laboratories, Faculty of Medical Sciences, Hodeidah University, Hodeidah, Yemen; Azal National Research Center, Azal University for Human Development, 447 Sana’a, Yemen; Department of Parasitology, Faculty of Medicine and Health Sciences, Sana’a University, 1247 Sana’a, Yemen; Department of Biomedical Science, Faculty of Medicine, University of Malaya, 50603 Kuala Lumpur, Malaysia

**Keywords:** Knowledge, Attitude, Practice, Schistosomiasis, Neglected tropical diseases, Yemen

## Abstract

**Background:**

Schistosomiasis is highly prevalent in Yemen, with an estimated 3 million cases, particularly among rural communities. This community-based study aims to evaluate the knowledge, attitude and practices (KAP) on schistosomiasis among rural communities in Yemen.

**Methods:**

A cross-sectional study was carried out among 250 households from ten rural districts in Yemen. Overall, 400 children were screened for urogenital and intestinal schistosomiasis. Moreover, parents were interviewed using a pre-tested questionnaire to collect information about the demographic and socioeconomic information and their KAP concerning schistosomiasis.

**Results:**

A total of 127 (31.8 %) children were found to be excreting schistosome eggs in either their urine or faeces (22.5 % *S. haematobium* and 8.0 % *S. mansoni*). Although 92.4 % of the respondents had heard about schistosomiasis, 49.8 %, 68.0 % and 47.2 % had knowledge concerning the transmission, signs and symptoms, and prevention, respectively. In addition, 77.1 % considered schistosomiasis as harmful while 48.5 % believed that schistosomiasis could be prevented, albeit their practices to prevent infections were still inadequate. Significant associations between the KAP and age, education, employment status and household monthly income were reported (*P* < 0.05). Moreover, a significantly higher level of knowledge was reported among the respondents who had infected children compared to those with no infected family members (*P* < 0.05). Multiple logistic regression analysis revealed that the level of education and the history of schistosomiasis were the most important factors associated with the KAP concerning schistosomiasis among this population.

**Conclusion:**

This study reveals that knowledge about the cause, transmission, symptoms and prevention of schistosomiasis among the rural population in Yemen was inadequate, and that this could be a challenging obstacle to the elimination of schistosomiasis in these communities. Besides the current mass drug administration, school and community-based health education regarding schistosomiasis is imperative among these communities to significantly reduce the transmission and morbidity of schistosomiasis.

## Background

Schistosomiasis is one of the most serious and prevalent neglected tropical diseases (NTDs) in many developing countries, particularly in Africa (which has about 90 % of the world’s reported cases), Latin America and the Middle East. Recent estimates revealed that more than 200 million people are infected with *Schistosoma* species worldwide, and that almost 700 million people are at risk of this infection [1, 2]. The disease is caused by different schistosoma species, with *S. haematobium* (causes urinary schistosomiasis), *S. mansoni* and *S. japonicum* (causes intestinal schistosomiasis) being the main and most common species. *S. haematobium* infection is characterized by haematuria as the classical sign. In chronic cases, bladder and ureteral fibrosis, sandy patches in the bladder mucosa and hydronephrosis occur while in advanced cases the infection is associated with bladder cancer [3–5]. Meanwhile, *S. mansoni* infection in humans causes diarrhoea, abdominal pain and blood in faeces. In the late stage, hepatosplenomegaly is the common complication with ascites and portal hypertension.

In December 2009, Yemen launched a six-year project to eliminate schistosomiasis-related morbidity and control intestinal worms throughout Yemen with financial support from the World Bank and WHO [6]. Referring to the funding agreement signed by the Ministry of Public Health and Population, Yemen, mass drug administration (MDA) was considered as the only intervention against schistosomiasis in Yemen [7]. However, health education materials (leaflets and posters) providing information on the transmission cycle and prevention of schistosomiasis and other helminth infections were also distributed during MAD campaigns [8]. The programme was disrupted by civil and political unrest resulting from “the Arab Spring” during 2010–2011 and re-commenced in 2012. Nonetheless, the current widespread civil war, which started in March 2015, has created more obstacles for the sustainability of the programme.

Despite the intensive control efforts by the government and international bodies, schistosomiasis is still a life-threatening public health problem in Yemen, with an estimated 3 million cases, which places the country second, after Egypt with 7 million cases, as having the highest prevalence of schistosomiasis in the Middle East [2, 9, 10]. Moreover, a recent study from Yemen showed that 59 % of the squamous cell carcinoma (SCC) cases were caused by *S. haematobium* chronic infection among adults [11]. However, data on the knowledge, attitude, and practices (KAP) of populations in endemic areas in Yemen are not available. Community awareness and involvement are considered as one of the cardinal tools for the success and sustainability of any disease control programme [12]. In low socioeconomic communities, intervention through public awareness is often recommended as a first line of action to create the enabling environment for other strategies to thrive [13]. Moreover, such data are of great importance for identifying, designing and implementing effective community-based control interventions [14–16]. Within this context, the present study aims to evaluate the KAP towards schistosomiasis in Yemen. It is hoped that our findings will provide new information about the schistosomiasis-related KAP of the targeted population, and will add new insights about the prevention and control of this devastating disease in Yemen.

## Methods

### Ethical consideration

The present study was carried out according to the guidelines laid down in the Declaration of Helsinki and all procedures involving human subjects were approved by the Medical Ethics Committee of the University of Malaya Medical Centre, Malaysia (reference number: 968.4). The protocol was also approved by the University of Hodeidah, and permission was also given by the Yemen Schistosomiasis National Control Project and health offices of the relevant provinces. Before commencement of the study, information about the objectives of the study and the role of the participants were given to the invited people. Moreover, they were informed that their participation was voluntary and that they could withdraw from the study at any stage without any consequences. Subsequently, written and signed or thumb-printed informed consent was obtained from the heads of the households to conduct the study and also on behalf of their children; these procedures were approved by the Medical Ethics Committee of the University of Malaya. All the infected children were treated with a single dose of 40 mg/kg body weight praziquantel tablet.

### Study design and study area

Being one of the poorest countries in the Middle East, Yemen has the highest percentage of people living in poverty with more than 50 % of the population of nearly 25 million people being estimated to be under the national poverty line [17]. The country has been unstable for several years, suffering from civil wars, a deteriorating economy and severe depletion in water resources, with only 25 % of the population having easy access to safe water.

**Fig. 1:**
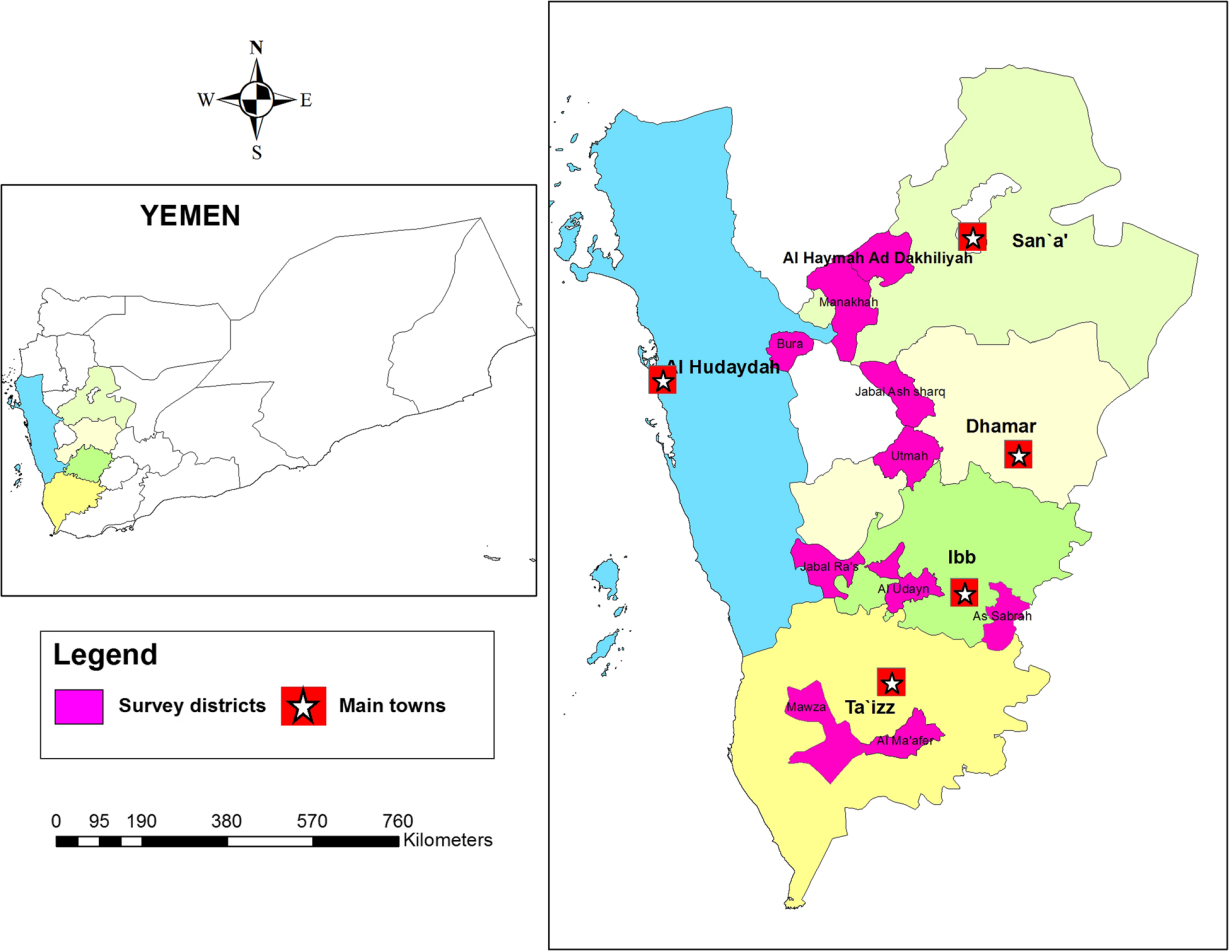
A geographic map showing the study area in Yemen (ten districts within five provinces)

A cross-sectional community-based study was conducted among the rural population in five different provinces in Yemen, namely Taiz, Ibb, Dhamar, Sana’a and Hodiedah. A questionnaire survey and sample collection was carried out between January and July 2012. In each province, two rural districts were randomly selected from the available official district list and then two villages within the selected districts were considered in collaboration with the Schistosomiasis Control Project office in each province (Fig. 1). The inclusion criteria in selecting these study areas were rural areas, > 50 households, undergoing active control surveillance and people’s willingness to participate in the study. The districts were Mosa and Almafer (Taiz), Alsabrah and Alodien (Ibb), Otmah and Gabal al sharq (Dhamar), Alhemah and Manakhah (Sana’a), and Gabal Ras and Bora (Hodiedah). These areas were known to be endemic for schistosomiasis and undergoing active surveillances by the schistosomiasis national control project. The number of inhabitants per household was recorded and all were invited to participate in this study. Unique reference codes were assigned to each household and study participant.

### Study population

Out of 780 households in the study areas, 250 households were randomly selected for this study (25 households from each village). At the village setting, a simple map was drawn for the distribution and location of houses and then every second house was selected for data collection. Of 632 children who were available in the selected houses and agreed to participate, a total of 430 (68.0 %) children aged ≤ 15 years delivered the containers for examination. Birth dates were obtained from the children’s birth certificates or immunization records (available for most of those aged below 10 years) or from any related official document available from the parents. In this study, 202 (32.0 %) failed to submit samples and/or were absent during the questionnaire survey, and 30 (4.7 %) containers were returned empty. Hence, they were excluded from the study. Overall, 400 (63.3 %) children (59.5 % males and 40.5 % females) who delivered suitable samples for examination together with complete questionnaire data were included in this study.

### Questionnaire survey

A validated questionnaire was used to collect data about the demographic, socio-economic, environmental background, personal hygiene, water contact and collection practices, and history of receiving anti-schistosomal treatment. Moreover, the questionnaire also involved questions concerning the knowledge about schistosomiasis aetiology, transmission, clinical manifestations, prevention and control. Trained research assistants collected information from the children’s parents via face-to-face interviews. The survey gathered information on both spontaneous and probed knowledge about schistosomiasis. Questions on knowledge were open-ended questions, without multiple-choice answers to avoid guessing, which might have given a false impression concerning the knowledge of the participant. However, the questions pertaining to the practices were provided with multiple-choice answers to assess the frequency of performing these activities or actions. At the end of each interview session, the interviewer probed for further knowledge related to schistosomiasis and not mentioned spontaneously by the respondent. However, this was made on selected variables only, specifically the source of information about the disease, history and reasons for previous visits to clinics or hospitals, reasons for not swimming in open water, and the identification of snails and whether or not they are harmful upon showing them to the respondents. The questionnaire was prepared in English and then translated into Arabic (the native language of Yemen). During the survey, direct observations were made concerning the cleanliness of the houses (such as availability of functioning toilets, washing soap, piped water, water containers and cisterns), and the personal hygiene of children (such as nail cutting, clothes, clean hands, wearing shoes when going outside the house).

### Parasitology

Faecal and urine samples were collected from children in 100 ml clean, labelled, and screw-capped containers. The samples were collected between 9–11 a.m. and transported within 5 h to the nearest laboratory in suitable cool boxes (4–6 °C). The faecal samples were examined using formalin ether sedimentation and Kato-Katz techniques for the presence of *S. mansoni* eggs [18]. Meanwhile, the urine samples were examined using the dipstick test and sedimentation method using a Nuclepore membrane for the presence of *S. haematobium* eggs [19]. To determine the worm burden, egg counts were taken and recorded as eggs per gram of faeces (EPG) for each positive sample and the intensity of infections was graded as heavy (≥400 EPG), moderate (100–399 EPG) or light (1–99 EPG) according to the criteria proposed by the WHO [20]. Similarly, egg counts were taken and recorded as eggs/10 μl urine (EP10ml), and the intensity of the infection was graded as heavy (>50 EP10ml) or light (1–50 EP10ml) [20]. For quality control, 25 % of the samples were selected randomly and re-examined by another assistant to confirm the results.

### Data analysis

Data were double entered by two different researchers into Microsoft Office Excel 2007 spreadsheets. Then, a third researcher crosschecked the two datasets for accuracy and created a single dataset. Data analysis was performed using IBM SPSS Statistics, version 18.0 (IBM Corporation, NY, USA). For descriptive analysis, the percentage was used to express the prevalence of infections and proportion of variable categories. Although the egg counts were found to be not normally distributed, there are biological justifications for using the arithmetic mean (± standard deviation, SD) rather than the median or geometric mean to express the egg count of each *Schistosoma* species [21]. Pearson’s Chi Square (χ^2^) test or Fisher’s exact test was used where appropriate to examine the difference in proportions between groups and to test the association between KAP and the demographic, socioeconomic and infection status. Odds ratios (ORs) and 95 % confidence intervals (CIs) were computed for all variables and *P* < 0.05 was considered as the level of significance. Multiple logistic regression analysis was performed to identify the factors significantly associated with the KAP variables among the studied population. A *P* value of < 0.05 was considered to be statistically significant.

## Results

In total, 250 Yemeni householders with a mean age of 42 ± 4.3 years were interviewed face-to-face to fill in the questionnaire on their KAP towards schistosomiasis (Table 1). Due to cultural customs, the majority (78.1 %) of the respondents were males, and more than half of the fathers had at least primary education (6 years of formal education). About two-thirds of the participants had low family monthly income (< YER20, 000; US$1 = YER 216). Less than one-third (30.9 %) of the houses had a piped water supply while about one quarter of the houses had electricity (mainly during night-time).

**Table 1:** Demographic and socioeconomic characteristics of the subjects who participated in the study (*n* = 250)

Characteristics	n (%)
Age groups	
< 40 years	114 (45.4)
≥ 40 years	136 (54.6)
Gender	
Males	195 (78.1)
Females	55 (21.9)
Socioeconomic status	
Educated fathers (at least primary)	141 (56.3)
Low household income (<YER20, 000)	157 (62.9)
Farmers and non working fathers	142 (57.0)
Large family size (>7 members)	124 (49.5)
Piped water supply	77 (30.9)
Electricity	72 (28.9)
Presence of toilet in house	137 (54.9)
History of schistosomiasis	126 (50.5)

All values are number (%). YER, Yemen Riyal; (US$1 = YER216)

### Prevalence and distribution of schistosomiasis

Of the 400 participants, 127 (31.8 %) were egg-positive for schistosomiasis. Overall, 90 participants (22.5 %) had urogenital schistosomiasis, 32 (8.0 %) had intestinal schistosomiasis and 5 (1.3 %) were co-infected with both *S. haematobium* and *S. mansoni*. Of the 95 *S. haematobium*-positive samples, 21 (21.1 %) were of heavy intensity with a mean EP10ml of 340 (±244) eggs while 74 (77.9 %) cases were of light intensity with a mean EP10ml of 17 (±10) eggs. Likewise, 15 (40.5 %) and 3 (8.1 %) *S. mansoni* cases were of moderate and heavy intensity with a mean of EPG of 212 (±82) and 637 (±93) eggs, respectively. Moreover, 19 (51.4 %) *S. mansoni* cases were light infections with a mean EPG of 50 (±23) eggs. Data on the prevalence, distribution and key factors significantly associated with schistosomiasis have been published previously in Sady et al. [10].

### Knowledge about schistosomiasis, its signs and symptoms, transmission and prevention

The results about the knowledge of the participants regarding schistosomiasis transmission, signs and symptoms and prevention are shown in Table 2. It was found that a majority of 231 (92.4 %) of the respondents had heard about schistosomiasis (locally known as Bilharsia). Moreover, 68.0 % (157/231) mentioned at least one sign or symptom related to the disease, of whom 92 (39.8 %) mentioned haematuria, 90 (39.0 %) mentioned abdominal pain, and 41 (17.7 %) mentioned blood in stools; 32.0 % could not cite any symptom.

**Table 2:** Knowledge about schistosomiasis, symptoms, transmission and prevention among rural population in Yemen who had prior knowledge on schistosomiasis (*n* = 231)

Variable	Number	Percent
Source of information		
Health centre for schistosomiasis control	154	66.8
Health clinic/hospitals	50	21.7
Mass media	5	2.2
School	4	1.7
Do not remember	18	7.6
Signs and symptoms		
Haematuria	92	39.8
Abdominal pain	90	39.0
Diarrhoea	83	35.9
Fever	64	27.7
Burning urination	42	18.2
Blood in stool	41	17.7
Vomiting	35	15.2
Itching	34	14.7
Pale face (anaemia)	33	14.3
Fatigue	33	14.3
Loss of appetite	31	13.4
Cough	28	12.1
Swollen abdomen	25	10.8
Dysentery	17	7.4
Do not know	74	32.0
Transmission		
Playing with soil	76	32.9
Swimming/bathing in infested water	66	28.6
Dirty hands	56	24.2
Eating contaminated food	55	23.8
Drinking untreated water	34	14.7
Snail	26	11.3
Do not know	116	50.2
Prevention		
Avoid playing with soil	72	31.2
Washing hands before eating	65	28.1
Avoid swimming/bathing in ponds/streams	62	26.8
Washing vegetables/fruit before eating	53	22.9
Taking anti-schistosomal drugs	43	18.6
Avoid drinking untreated water	33	14.3
Avoid washing clothes in ponds/streams	24	10.4
Do not know	122	52.8

The results further showed that there was a lack of knowledge about the transmission of schistosomiasis among these people in that only 49.8 % (115/231) of the respondents were able to mention at least one mode of transmission. Of these, 28.6 % correlated the disease with contact with infested water while 11.3 % (26/231) indicated the role of snail vectors. Concerning the knowledge about the prevention of schistosomiasis, 47.2 % (109/231) were able to give at least one measure of prevention, with 26.8 % mentioning avoiding swimming in ponds/streams, 10.4 % avoiding washing clothes in ponds/streams, and 18.6 % mentioned taking anti-schistosomal drugs.

### Attitude and practices of people in rural Yemen to schistosomiasis

Table 3 shows the results about the attitude and practices of the respondents towards schistosomiasis. Of the participants who had prior knowledge concerning schistosomiasis, 77.1 % (178/231) considered schistosomiasis as a serious disease. Surprisingly, less than half (48.5 %) of the respondents believed that schistosomiasis can be prevented and only 13.0 % agreed about the role of faeces and urine as the source of infection. With regards to the practices of these people, the results showed that 58.8 % (147/250) of the respondents swim or bathe in open water and 59.2 % (148/250) practice open defaecation and/or urination in the water and fields. Likewise, 139 (55.5 %) of the respondents fetch water from ponds/streams/pumps/ wells and 83 (33.3 %) wash clothes in such water. In terms of treatment-seeking behaviour, 81.1 % (203/250) of the respondents mentioned that they go to the nearest clinic/hospital for treatment in case of gastrointestinal symptoms or haematuria while 4.5 % do nothing. Interestingly, 14.3 % (36/250) of the participants revealed that they go to traditional healers for the treatment of such signs and symptoms; however, none were able to mention the name of any medicinal plant or traditional remedy they had used.

**Table 3:** Attitude and practices of the participants towards schistosomiasis in rural Yemen

Variables	Number	Percent
Is schistosomiasis a serious disease? (*n* = 231)		
Yes	178	77.1
No	15	6.5
Do not know	38	16.4
Believes that schistosomiasis can be prevented (*n* = 231)		
Yes	112	48.5
No	87	37.7
Do not know	32	13.9
Faeces/urine as a source of infections (*n* = 231)		
Yes	30	13.0
No	26	11.2
Do not know	175	75.8
Practices (*n* = 250)		
Swimming/bathing in open water source	147	58.8
Indiscriminate defaecation/urination	148	59.2
Fetching water from ponds/streams	139	55.5
Washing clothes or utensil in open water source	83	33.3
Wearing shoes when going outside	186	74.3
Drinking untreated water	173	69.1
Seeking treatment from clinic for GIT and urinary tract symptoms	203	81.1
Seeking traditional treatment for GIT and urinary tract symptoms	36	14.3
Do nothing for GIT and urinary tract symptoms	11	4.5

### Association of knowledge of the participants about schistosomiasis with some demographic and socioeconomic factors

Table 4 shows that a significantly lower percentage of those aged below 40 years had heard about schistosomiasis compared to those aged ≥ 40 years (88.6 % vs. 95.6 %; *P* = 0.038). However, knowledge of fatigue (*P* = 0.021), swollen abdomen (*P* = 0.031) and vomiting (*P* = 0.001) as symptoms of schistosomiasis was significantly higher in those aged below 40 years compared to those aged ≥ 40 years. Similarly, the percentage of educated parents who had heard about schistosomiasis was significantly higher than that for non-educated parents (96.5 % vs. 87.2 %; *P* = 0.006). It was found that 73.8 % of the educated respondents had knowledge about at least one symptom of the disease compared to 49.1 % of their non-educated counterparts (*P* < 0.001). Likewise, respondents who had at least 6 years of formal education had a significantly higher level of knowledge about the role of snails as a mode of transmission of schistosomiasis (*P* = 0.036), and about avoiding swimming/playing in open water (*P* = 0.003), as well as about taking anti-schistosomal drugs as preventive measures (*P* = 0.001) compared to the non-educated respondents.

**Table 4:** Associations of knowledge of the participants about schistosomiasis with their age and educational level

Variable	Age (years)				Educational level			
	> = 40	<40	OR	95 % CI	Non educated	Educated	OR	95 % CI
Heard about schistosomiasis	130(95.6)	101(88.6)	0.4	0.1, 0.9*	95(87.2)	136(96.5)	4.0	1.4, 11.5*
Signs and symptoms:								
At least one symptom	88(64.7)	69(61.1)	0.9	0.5, 1.4	53(49.1)	104(73.8)	2.9	1.7, 4.9*
Haematuria	47(34.6)	45(39.8)	1.3	0.7, 2.1	30(27.5)	62(44.0)	2.1	1.2, 3.5*
Abdominal pain	52(38.2)	37(32.7)	0.8	0.5, 1.3	35(32.1)	55(39.0)	1.4	0.8, 2.3
Burning urination	22(16.2)	20(17.5)	1.1	0.6, 2.2	16(14.7)	26918.4)	1.3	0.7, 2.6
Blood in stool	26(19.0)	16(14.0)	0.7	0.4, 1.4	11(10.1)	30(21.4)	2.4	1.2, 5.1*
Itching	17(12.4)	17(14.9)	1.2	0.6, 2.6	12(11.0)	22(15.6)	1.5	0.7, 3.2
Pale face (anaemia)	15(10.9)	19(16.7)	1.6	0.8, 3.4	13(11.9)	20(14.3)	1.2	0.6, 2.6
Fatigue	11(8.0)	20(17.7)	2.5	1.1, 5.4*	14(12.8)	17(12.1)	0.9	0.4, 2.0
Swollen abdomen	9(6.6)	17(14.9)	2.5	1.1, 5.8*	10(9.2)	15(10.6)	1.2	0.5, 2.7
Diarrhoea	44(32.1)	39(34.5)	1.1	0.7, 1.9	25(22.7)	58(41.4)	2.4	1.4, 4.2*
Loss of appetite	19(13.9)	14(12.4)	0.9	0.4, 1.8	11(10.0)	22(15.6)	1.7	0.8, 3.6
Fever	35(25.5)	29(25.7)	1.0	0.6, 1.8	21(19.3)	43(30.5)	1.8	1.0, 3.3*
Vomiting	8(5.8)	23(20.4)	4.1	1.8, 9.6*	11(10.0)	21(14.9)	1.6	0.7, 3.4
Dysentery	11(8.0)	6(5.3)	0.6	0.2, 1.8	3(2.8)	13(9.2)	3.6	1.0, 12.9*
Transmission:								
At least one way of transmission	56(45.9)	59(45.7)	1.0	0.6, 1.6	45(41.3)	69(48.9)	1.4	0.8, 2.3
Swimming/bathing/playing in infested water	35(28.7)	31(24.2)	0.8	0.5, 1.4	24(22.0)	42(29.8)	1.4	0.8, 2.7
Snail	11(9.1)	14(10.9)	1.2	0.5, 2.8	6(5.5)	19(13.6)	2.7	1.1, 7.0*
Playing with soil	40(32.8)	37(28.9)	0.8	0.5, 1.4	29(26.6)	48(34.0)	1.4	0.8, 2.5
Dirty hands	27(22.3)	29(22.7)	1.0	0.6, 1.9	19(17.4)	38(27.0)	1.7	0.9, 3.2
Eating contaminated food	25(20.5)	31(24.2)	1.2	0.7, 2.3	19(17.3)	38(27.0)	1.8	0.9, 3.3
Drinking untreated water	16(13.1)	18(14.1)	1.1	0.5, 2.2	7(6.4)	26(18.6)	3.3	1.4, 8.0*
Prevention:								
At least one preventive measure	55(45.1)	58(45.3)	1.0	0.6, 1.7	35(31.8)	79(56.0)	2.7	1.6, 4.6*
Avoid swimming/playing in ponds/streams	38(31.1)	27(21.1)	0.6	0.3, 1.0	18(16.5)	47(33.3)	2.5	1.4, 4.7*
Avoid washing clothes in ponds/streams	12(9.8)	14(10.9)	1.2	0.5, 2.5	11(10.1)	15(10.6)	1.1	0.5, 2.4
Avoid playing with soil	39(32.0)	33(25.8)	0.7	0.4, 1.3	25(22.7)	47(33.3)	1.7	0.9, 3.0
Washing hands before eating	31(25.6)	35(27.3)	1.1	0.6, 1.9	22(20.0)	45(31.9)	1.9	1.1, 3.4*
Avoid drinking untreated water	14(11.5)	19(14.8)	1.3	0.6, 2.8	10(9.1)	23(16.4)	2.0	0.9, 4.3
Washing vegetables/fruit before eating	29(23.8)	24(18.8)	0.7	0.4, 1.4	18(16.5)	35(24.8)	1.7	0.9, 3.1
Taking anti-schistosomal drugs	21(17.4)	21(16.4)	0.9	0.5, 1.8	9(8.3)	33(23.6)	3.4	1.6, 7.5*

All values are number (%). OR, Odds ratio. CI, Confidence interval. *Significant association (*P* < 0.05)

Table 5 shows that the percentage of respondents who had heard about schistosomiasis was significantly higher among the working respondents (97.2 % vs. 88.7 %; *P* = 0.012) and those who had a household monthly income of ≥ YR20, 000 (97.8 % vs. 89.2 %; *P* = 0.012) when compared to the non-working residents and those who had low household monthly income. Likewise, the results revealed a significantly higher level of knowledge about the signs and symptoms among the working respondents and those with a household monthly income of ≥ YR20, 000 compared to their counterparts.

**Table 5:** Associations of knowledge of the participants about schistosomiasis with their employment status and household monthly income

Variable	Employment status				Household monthly income			
	Not working	Working	OR	95 % CI	<YR20,000	≥ YR20,000	OR	95 % CI
Heard about schistosomiasis	126(88.7)	105(97.2)	4.4	1.3, 9.6*	140(89.2)	91(97.8)	5.5	1.2, 24.5*
Signs and symptoms:								
At least one symptom	86(60.6)	71(66.4)	1.3	0.8, 2.2	97(61.8)	60(64.5)	1.1	0.7, 1.9
Haematuria	41(28.7)	51(47.7)	2.3	1.3, 3.8*	49(31.0)	43(46.7)	2.0	1.1, 3.3*
Abdominal pain	58(40.8)	31(29.0)	0.6	0.3, 1.0	62(39.5)	28(30.1)	0.7	0.4, 1.1
Burning urination	23(16.1)	19(17.8)	1.1	0.6, 2.2	25(15.9)	17(18.3)	1.2	0.6, 2.3
Blood in stool	26(18.3)	15(14.0)	0.7	0.4, 1.5	25(15.9)	16(17.2)	1.1	0.6, 2.2
Itching	18(12.7)	15(14.0)	1.1	0.5, 2.3	19(12.1)	14(15.2)	1.3	0.6, 2.7
Pale face (anaemia)	16(11.3)	17(15.6)	1.5	0.7, 3.1	20(12.7)	13(14.0)	1.1	0.5, 2.4
Fatigue	12(14.8)	1099.3)	0.6	0.3, 1.3	25(15.9)	6(6.5)	0.4	0.1, 0.9*
Swollen abdomen	13(9.2)	12(11.2)	1.3	0.5, 2.8	14(8.9)	11(11.8)	1.4	0.6, 3.2
Diarrhoea	39(27.5)	44(40.7)	1.8	1.1, 3.1*	43(27.4)	40(43.0)	2.0	1.2, 3.4*
Loss of appetite	12(8.4)	21(19.4)	2.6	1.2, 5.6*	15(9.5)	18(19.4)	2.3	1.1, 4.8*
Fever	36(25.2)	28(26.2)	1.1	0.6, 1.9	43(27.4)	21(22.6)	0.8	0.4, 1.4
Vomiting	16(11.3)	15(13.9)	1.3	0.6, 2.7	17(10.8)	14(15.2)	1.5	0.7, 3.2
Dysentery	1(0.7)	15(14)	13.0	3.0, 37.0*	6(3.8)	11(11.8)	3.4	1.2, 9.5*
Transmission:								
At least one way of transmission	60(42.0)	54(50.0)	1.4	0.8, 2.3	69(43.7)	45(48.9)	1.2	0.7, 2.1
Swimming / bathing / playing in infested water	32(22.5)	34(31.8)	1.6	0.9, 2.8	38(24.1)	29(31.2)	1.4	0.8, 2.5
Snail	14(9.9)	11(10.3)	1.0	0.5, 2.4	18(11.5)	8(8.6)	0.7	0.3, 1.7
Playing with soil	34(23.9)	43(39.8)	2.1	1.2, 3.6*	40(25.5)	37(39.8)	1.9	1.1, 3.3*
Dirty hands	28(19.6)	29(26.9)	1.5	0.8, 2.7	35(22.2)	22(23.7)	1.1	0.6, 2.0
Eating contaminated food	34(23.8)	23(21.3)	0.9	0.5, 1.6	39(24.8)	18(19.4)	0.7	0.4, 1.4
Drinking untreated water	14(9.9)	19(17.8)	1.9	0.9, 4.1	22(13.9)	12(12.9)	0.9	0.4, 1.9
Prevention:								
At least one preventive measure	62(43.7)	51(47.2)	1.2	0.7, 1.9	67(42.7)	46(49.5)	1.3	0.8, 2.2
Avoid swimming/playing in ponds/streams	28(19.7)	36(33.6)	2.1	1.2, 3.7*	37(23.6)	27(29.3)	1.3	0.8, 2.4
Avoid washing clothes in ponds/streams	15(10.5)	11(10.2)	1.0	0.4, 2.2	20(12.7)	5(5.4)	0.4	0.1, 1.1
Avoid playing with soil	33(23.2)	38(35.5)	1.8	1.0, 3.2*	38(24.2)	34(36.6)	1.8	1.0, 3.2*
Washing hands before eating	28(19.6)	39(36.1)	2.3	1.3, 4.1*	35(22.3)	31(33.3)	1.7	0.9, 3.1
Avoid drinking untreated water	15(10.6)	18(16.7)	1.7	0.8, 3.5	16(10.2)	17(18.3)	1.9	0.9, 4.1
Washing vegetables/fruit before eating	29(20.4)	24(22.2)	1.1	0.6, 2.0	34(21.5)	19(20.7)	0.9	0.5, 1.8
Taking anti-schistosomal drugs	19(13.4)	23(21.3)	1.7	0.9, 3.4	19(12.1)	24(25.8)	2.5	1.3, 4.9*

All values are number (%). OR, Odds ratio. CI, Confidence interval. *Significant association (*P* < 0.05)

### Association of attitude and practices of the participants towards schistosomiasis with some demographic and socioeconomic factors

Tables 6 and 7 show a significant association of attitude towards schistosomiasis with the age of the respondents (*P* = 0.030), while the association of attitude with the educational level, employment status and household monthly income of the respondents was not significant (*P* > 0.05). Moreover, practising indiscriminate defaecation/urination (66.7 % vs. 52.9 %; *P* = 0.028) and clothes or utensil washing in open water (41.6 % vs. 26.3 %; *P* = 0.010) were found to be significantly higher in the respondents aged below 40 years than those aged ≥ 40 years (Table 6). On the other hand, practising indiscriminate defaecation/urination was significantly lower among the respondents who were educated (44.3 % vs. 78.2 %; *P* < 0.001), working (37.4 % vs. 75.5 %; *P* < 0.001) and with a household monthly income of ≥ YR20, 000 (30.1 % vs. 76.4 %; *P* < 0.001) compared to their counterparts.

**Table 6:** Associations of attitude and practices of the participants towards schistosomiasis with their age and educational level

Variable	Age (years)				Educational level			
	> = 40	<40	OR	95 % CI	Non educated	Educated	OR	95 % CI
Attitude:								
Serious disease	108(82.4)	71(70.3)	0.5	0.3, 0.9*	71(74.7)	107(79.3)	1.2	0.7, 2.4
Practices:								
Swimming/bathing in open water source	34(24.8)	33(28.9)	1.2	0.7, 2.2	24(22.0)	42(29.8)	1.5	0.8, 2.7
Indiscriminate defaecation/urination	72(52.9)	76(66.7)	1.8	1.1, 3.0*	86(78.2)	62(44.3)	0.2	0.1, 0.4*
Fetching water from ponds/streams	70(51.1)	69(61.1)	1.5	0.9, 2.5	58(53.2)	81(57.4)	1.2	0.7, 2.0
Washing clothes & utensil in open water	36(26.3)	47(41.6)	2.0	1.2, 3.4*	35(32.1)	48(34.0)	1.1	0.6, 1.9
Wearing shoes when go outside	100(73.5)	85(75.2)	1.1	0.6, 1.9	80(73.4)	106(75.2)	1.1	0.6, 1.9
Drinking untreated water	96(70.6)	77(67.5)	0.9	0.5, 1.5	83(75.5)	90(63.8)	0.6	0.3, 0.9*
Seeking treatment from clinic for GIT and urinary tract symptoms	109(79.6)	94(83.2)	1.3	0.7, 2.4	84(76.4)	119(85.0)	1.8	0.9, 3.3

All values are number (%). OR, Odds ratio. CI, Confidence interval. *Significant association (*P* < 0.05)

**Table 7:** Associations of attitude and practices of the participants towards schistosomiasis with their employment status and household monthly income

Variable	Employment status				Household monthly income			
	Not working	working	OR	95 % CI	<YR20,000	≥ YR20,000	OR	95 % CI
Attitude:								
Serious disease	92(73.0)	86(82.7)	1.8	0.9, 3.4	105(75.0)	73(80.2)	1.4	0.7, 2.6
Practices:								
Swimming/bathing in open water source	32(22.5)	34(31.8)	1.6	0.9, 2.8	38(24.1)	29(31.2)	1.4	0.8, 2.5
Indiscriminate defaecation/urination	108(75.5)	40(37.4)	0.2	0.2, 0.3*	120(76.4)	28(30.1)	0.1	0.1, 0.2*
Fetching water from ponds/streams	78(54.4)	61(57.0)	1.1	0.7, 1.8	88(55.7)	51(54.8)	1.0	0.6, 1.6
Washing clothes & utensil in open water	41(28.7)	43(39.8)	1.6	0.9, 2.8	47(29.7)	36(39.1)	1.5	0.9, 2.6
Wearing shoes when outside	81(57.0)	105(97.2)	19.4	8.0, 47.1*	100(63.7)	86(92.5)	7.0	3.0, 16.2*
Drinking untreated water	100(70.4)	73(67.6)	0.9	0.5, 1.5	108(68.8)	65(69.9)	1.1	0.6, 1.8
Seeking treatment from clinic for GIT and urinary tract symptoms	110(77.5)	93(86.1)	1.8	0.9, 2.5	124(79.0)	79(84.9)	1.5	0.8, 3.0

All values are number (%). OR, Odds ratio. CI, Confidence interval. *Significant association (*P* < 0.05)

### Association of KAP of the participants on schistosomiasis with the infection status among their children

The association between the knowledge of participants on schistosomiasis (prior knowledge concerning the signs and symptoms, transmission and prevention) and the infection status among their children was investigated. The results showed that the knowledge about haematuria (*P* = 0.042), burning urination (*P* = 0.045) and pale face (anaemia) (*P* = 0.017) as signs and symptoms of schistosomiasis, and avoiding washing clothes in open water sources as a preventive measure (*P* = 0.12) was significantly higher among those who had infected children compared to those with no infected family members. Likewise, a higher level of knowledge about blood in stools as a symptom (23.4 % vs. 15.6 %), and swimming or bathing in open water sources (13.0 % vs. 6.5 %) and snails (15.8 % vs. 8.4 %) as modes of transmission of schistosomiasis was reported among the respondents who had infected children compared to those with non-infected children; however, the difference was not statistically significant (*P* > 0.05).

### Multivariate analysis

The results of multiple logistic regression analyses for the factors significantly associated with the KAP on schistosomiasis among the Yemeni participants showed that the educational level of the respondents was the most important factor as it was significantly associated with many KAP items. The results indicated that respondents who had at least 6 years of formal education had significantly higher odds of hearing about schistosomiasis (OR = 3.84; 95 % CI = 1.25, 11.81), had knowledge about the signs and symptoms (OR = 3.37; 95 % CI = 1.89, 6.01), had knowledge about the presence of blood in stools (OR = 3.66; 95 % CI = 1.60, 8.38) and diarrhoea (OR = 2.06; 95 % CI = 1.12, 3.78) as symptoms of schistosomiasis; had knowledge concerning the role of snails in the transmission of schistosomiasis (OR = 3.91; 95 % CI = 1.41, 10.91); had knowledge about the prevention of schistosomiasis (OR = 1.85; 95 % CI = 1.04, 3.28); and had knowledge about taking anti-schistosomal drugs as a preventive measure (OR = 3.17; 95 % CI = 1.36, 7.39) when compared with non-educated respondents.

Logistic regression analyses also showed that respondents who had a history of schistosomiasis showed higher odds of having knowledge about a swollen abdomen (OR = 4.39; 95 % CI = 1.50, 12.84) and anaemia (OR = 2.73; 95 % CI = 1.10, 6.75) as symptoms; swimming or playing in open water sources as a mode of transmission (OR = 2.77; 95 % CI = 1.24, 6.17); and had knowledge about the prevention of schistosomiasis (OR = 2.42; 95 % CI = 1.31, 4.47) when compared with those with no previous history of infection. Moreover, respondents aged below 40 years were less likely to hear about schistosomiasis (OR = 0.31; 95 % CI = 0.10, 0.78).

## Discussion

The present study is the first to provide information about the knowledge, attitude and practices (KAP) concerning schistosomiasis in Yemen. Despite the intensive efforts to control the disease, schistosomiasis is still highly prevalent among children in rural Yemen and our previously published work revealed that 31.8 % of the participants were infected with *Schistosoma* species [10]. It was found that the overall prevalence was 22.5 % for *S. haematobium*, 8.0 % for *S. mansoni*, and 1.3 % for co-infection with both *S. haematobium* and *S. mansoni*. Although the majority of the respondents had heard about schistosomiasis, the results showed that awareness about the symptoms, ways of transmission and preventive measures among the participants was generally poor. The present study was carried out in endemic areas that are undergoing active control and prevention surveillance by the schistosomiasis national control project (SNCP), which may explain why 92.4 % of the respondents had heard about the disease. This is also consistent with the finding that half of the respondents declared a history of infection among their children, which supports the endemicity of infection in these communities. This was also supported by the significantly higher level of schistosomiasis-related knowledge among respondents who had infected children. Our findings showed that two-thirds of the respondents indicated that they had heard about the disease from the control programme personnel while 21.7 % cited the clinics and hospitals as the source of their information. Moreover, the present study revealed poor contribution from the media and schools in Yemen, which should receive proper attention as involvement of the media and schools is imperative in the battle against this devastating disease.

Overall, these findings are in agreement with previous studies from other schistosomiasis-endemic countries; a high level of awareness of schistosomiasis has been reported in Brazil [22], Egypt [23] and Kenya [24]. In contrast, poor awareness about schistosomiasis has been reported in Malawi [25], Zimbabwe [15] and Western Kenya [26]. Despite seven years of health education interventions using a diversity of communication outlets including radio, television and posters, a previous study in Senegal revealed that although 86 % of the respondents stated that they had heard about schistosomiasis, only 30 % had adequate knowledge about the symptoms and modes of transmission of the disease [27].

The present study revealed poor knowledge about the modes of transmission and preventive measures of schistosomiasis; particularly the role of snails in the transmission of schistosomiasis. When the snails were shown to the respondents, most of them declared that there are plenty of these creatures in the streams, ponds and irrigation canals and also mentioned that “snails are not harmful and usually our children like to collect and play with them”. In western Kenya, a previous study found that some of the participants knew that snails and poor sanitation contributed to the spread of the disease, but lacked understanding of the transmission cycle [26]. Hence, it is clear that the lack of this knowledge among the targeted population may create an additional burden and cost for controlling the disease and may cause the failure of the schistosomiasis eradication programme.

Our findings revealed that one-third of the respondents could not associate the infection with any symptom. Conversely, previous studies from Brazil and Ethiopia reported diverging information where the majority of the subjects were able to associate these symptoms with the infection [22, 28]. Similarly, it is also worth noting that knowledge about the symptoms of intestinal schistosomiasis among the respondents was negligible, as only 17.7 % of them mentioned blood in stools. This could be attributed to the disease being frequently confused with other intestinal infections exhibiting similar symptoms, such as amoebic dysentery, which is common among the targeted populations [29, 30]. Another reason could be the lower prevalence of intestinal schistosomiasis compared to urogenital schistosomiasis.

In respect of treatment-seeking behaviour, the majority of the participants seek treatment for haematuria and GIT symptoms at health centres. However, time is a major concern as all such participants acknowledged that there is always a delay in seeking treatment because many people do not seek care for symptoms until they become severe. Poverty contributes significantly to this delay, as the patients may not earn enough to pay for the cost of transport and medical services. In addition, infected individuals may only decide to seek treatment from health centres when they cannot tolerate the symptoms of the disease any longer and they receive treatment at a late stage of the disease. This is especially detrimental considering that morbidity in schistosomiasis is a function of the infection intensity and duration. In addition, this could also be attributed to the poor knowledge about the fatal complications of schistosomiasis [24]. Furthermore, the present study showed that 14.3 % of the respondents thought that treating schistosomiasis at a health centre is very expensive and cited this as a reason for seeking alternative treatment. Similar findings and cited reasons were reported in Western Kenya and Uganda [24, 31]. Previous studies in Ghana showed that more than 70 % of the schistosomiasis-infected individuals opt for traditional self-treatment without visiting a health facility, with ‘Do not have the money’ and ‘Not serious enough’ being the commonest reasons for not visiting a clinic [32, 33].

It should be noted that a considerable number of participants in these communities showed confusion between the schistosomiasis and soil-transmitted helminth (STH) infections, which are also endemic in these areas. This could be attributed to the practice of the MDA that was carried out by the SNCP 2010–2016, which covered both diseases. However, this may lead to serious complications in patients who believe that schistosomiasis can be cured by taking albendazole and could contribute to the transmission of the disease within endemic communities. Similarly, some of the respondents questioned the usefulness of the drug as it cannot prevent them from reinfection. For this reason, 11 respondents (four males and seven females, aged > 50 years) declared that they were suffering with haematuria but do nothing about its treatment.

Our study also reported many mistaken beliefs about schistosomiasis as a large number of the respondents demonstrated misconceptions about the transmission and prevention of schistosomiasis. For instance, a noteworthy number of participants believed that schistosomiasis is transmitted by playing with soil, eating contaminated food and from dirty hands. Likewise, similar percentages of the respondents mentioned avoiding playing with soil, washing hands before eating and washing vegetables/fruits before consumption for the prevention of schistosomiasis. Since 2006, Praziquantel has been distributed together with albendazole tablets to protect against STH infections, which are highly endemic in these areas [34]. When questioned about the history of deworming, many of the respondents described the two orange flavoured tablets (albendazole).

Interestingly, our findings showed that less than half of the participants believed that schistosomiasis is a preventable disease, while one third of them believed that acquiring schistosomiasis could not be prevented. Besides poor health education, this could be due to continuous exposure and reinfection after repeated deworming in these communities. Furthermore, our findings showed that only 13 % of the respondents knew that faeces and urine are the sources of infection while three-quarters did not know. This indicates a lack of health education about the causes and prevention of schistosomiasis among these people, which should be provided during the mass drug administration campaigns.

In investigating the factors associated with the respondents’ KAP, we found that educated, working and those who had ≥ YR20, 000 monthly income had significantly better knowledge about the signs and symptoms, transmission (snail) and prevention of schistosomiasis when compared to their counterparts. Education plays an important role in people’s perceptions and practices of controlling schistosomiasis [35]. Previous studies from Africa and Asia showed that the odds of having a lower knowledge about schistosomiasis were significantly higher in the respondents who had a primary education level or below [36, 37]. In agreement with these findings, our study showed a significant impact for the level of education on the population’s KAP concerning schistosomiasis. This could be attributed to the ability of reading and understanding the leaflets and posters distributed by the control programme. In contrast, previous studies from Cote d’Ivoire and Uganda found no significant association between the educational level and the level of knowledge of schistosomiasis [16, 31]. Our results showed that only four respondents declared schools as the source of information about schistosomiasis, which needs attention, as the school can play an important role in controlling different diseases among the targeted communities. This can be done through health promoting programmes that teach the students how to protect themselves against infections and then disseminate information to the surrounding communities [38, 39].

In respect of gender, our findings revealed a similar level of knowledge and attitude towards schistosomiasis between the male and female respondents. This is in accordance with our previous findings that there was no significant difference in the prevalence of schistosomiasis among males and females [10]. However, previous studies from Malawi, Zanzibar and South Darfur reported a significantly higher prevalence of infection and better knowledge about the disease among male participants compared to females [40–42]. This could be due to some religious and cultural beliefs. In Islamic communities, females are not allowed to swim or bathe in the open water sources and are not allowed to participate in fishing and irrigation activities [43, 44]. However, in rural Yemen, females are responsible for fetching water and washing clothes and utensils at streams, ponds, dams and other water sources, as was observed in the study area [10]. It is well known that poverty propagates the occurrence of many health problems including NTDs in developing communities. Within this context, our findings revealed a significantly higher level of knowledge and attitude towards schistosomiasis among respondents who had a household monthly income of ≥ YR20, 000 compared to those with a low income.

Recently, the WHO called for a global effort to eliminate human schistosomiasis by 2025, with MDA as a cardinal intervention [45]. However, this call has underscored the need for more emphasis on snail-related research [46]. Despite the potential threat of drug resistance developing in the schistosomes [47], MDA has been used for generations as the main pillar and the most cost-effective intervention to control schistosomiasis; however, chemotherapy alone may never achieve transmission control or elimination [48, 49]. Thus, other interventions, such as snail control and health education among schistosomiasis-endemic communities are essential and should be implemented in parallel with MDA [50, 51]. Adema et al*.* [46] raised some issues pertinent to snail control, such as a decline in snail related funding and a concomitant decline, to a shockingly low level, as well as the availability of investigators and young workers who are able to identify medically relevant snails. Great success has been achieved in many countries, such as China and Egypt, in reducing the transmission, prevalence and morbidity of schistosomiasis [36, 52, 53]. The MDA-based strategy was fundamental in this success hand-in-hand with health education campaigns and capacity building through the training of personnel working in rural health units or involved in reducing the roles of humans and animals as sources of infection for snails [52, 53]. Similar success can also be achieved in Yemen through an integrated national control approach that should consider the MDA, snail control and health education. Rural communities in Yemen share similar socioeconomic and health profiles with a different climate. Coastal plains and foothills (Taiz, Ibb and Hodeidah) have more streams, whereas mountainous areas (Sana’a and Dhamar) have more water pools/troughs and dams. Thus, we believe that our findings are generalisable to the entire rural population in Yemen. However, further studies in other provinces are required to confirm this conjecture.

## Conclusion

This study reveals inadequate knowledge, attitude and practices concerning schistosomiasis among the rural population residing in schistosomiasis endemic areas in Yemen, which could be a challenging obstacle to the endeavour towards the elimination of schistosomiasis from Yemen. The study also shows an alarmingly high prevalence of schistosomiasis among children in these areas. Thus, there is a great need for a proper health education intervention and community mobilisation in order to enhance prevention and instil better knowledge concerning the transmission and prevention of schistosomiasis. Providing efficient health education to people residing in schistosomiasis endemic areas is imperative for an effective and sustainable control programme in order to save the lives and future of the most vulnerable children in rural Yemen.
